# Molnupiravir Inhibits Replication of Multiple *Alphacoronavirus suis* Strains in Feline Cells

**DOI:** 10.3390/pathogens14080787

**Published:** 2025-08-07

**Authors:** Tomoyoshi Doki, Kazuki Shinohara, Kaito To, Tomomi Takano

**Affiliations:** Laboratory of Veterinary Infectious Disease, School of Veterinary Medicine, Kitasato University, Towada 034-8628, Aomori, Japan; doki@vmas.kitasato-u.ac.jp (T.D.); to.kaito@st.kitasato-u.ac.jp (K.T.)

**Keywords:** molnupiravir, *Alphacoronavirus suis*, cross-species transmission, antiviral activity, One Health

## Abstract

The cross-species spillover of coronaviruses is considered a serious public health risk. Feline coronavirus (FCoV), canine coronavirus (CCoV), and transmissible gastroenteritis virus (TGEV) are all classified under *Alphacoronavirus suis* and infect companion animals and livestock. Due to their frequent contact with humans, these viruses pose a potential risk of future cross-species transmission. Molnupiravir, a prodrug of N4-hydroxycytidine, exhibits potent antiviral activity against SARS-CoV-2, a member of the *Betacoronavirus* genus, and has been approved for the treatment of COVID-19. Molnupiravir was recently shown to be effective against FCoV, suggesting broad-spectrum antiviral activity across coronavirus lineages. Based on these findings, the present study investigated whether molnupiravir is also effective against CCoV and TGEV, which belong to the same *Alphacoronavirus suis* species as FCoV. We examined the in vitro antiviral effects of molnupiravir using four viral strains: FCoV-1 and -2, CCoV-2, and TGEV. Molnupiravir inhibited plaque formation, viral antigen expression, the production of infectious viral particles, and viral RNA replication in a dose-dependent manner in all strains. IC_50_ values for CCoV-2 and TGEV, calculated using a feline-derived cell line (fcwf-4), were significantly lower than those for FCoV, suggesting higher sensitivity to molnupiravir. These results demonstrate that molnupiravir exhibited broad antiviral activity against animal coronaviruses classified under *Alphacoronavirus suis*, providing a foundation for antiviral strategies to mitigate the future risk of cross-species transmission.

## 1. Introduction

Coronaviruses are enveloped, positive-sense, single-stranded RNA viruses that encode four essential structural proteins: the spike, envelope, membrane, and nucleocapsid (N) proteins. Some lineages also express an additional hemagglutinin esterase protein [1,2]. According to the 2024 taxonomy release by the International Committee on Taxonomy of Viruses [3], coronaviruses are classified within the order *Nidovirales*, family *Coronaviridae*, subfamily *Orthocoronavirinae,* and are subdivided into four genera: *Alphacoronavirus*, *Betacoronavirus*, *Gammacoronavirus*, and *Deltacoronavirus* [3]. Of these, *Alphacoronavirus* and *Betacoronavirus* predominantly comprise species that infect mammalian hosts.

The species *Alphacoronavirus suis* within the genus *Alphacoronavirus* includes animal coronaviruses that infect companion animals and livestock. This species comprises feline coronavirus (FCoV), canine coronavirus (CCoV), and swine transmissible gastroenteritis virus (TGEV). Both FCoV and CCoV are classified into two serotypes [4,5]. FCoV typically causes mild enteritis in felids but can occasionally lead to a fatal inflammatory disease known as feline infectious peritonitis (FIP) [6,7]. CCoV is associated with enteritis in dogs, particularly in puppies, where it can contribute to gastrointestinal disease [8]. While infections are often subclinical in adult dogs, severe cases have been reported in young animals, especially when co-infected with other pathogens. Notably, a pantropic and highly virulent strain of CCoV has been identified, which can cause multisystemic disease and marked leukopenia [9,10]. TGEV is the causative agent of transmissible gastroenteritis in pigs [11]. Although the disease is generally mild in adult pigs, it causes acute gastroenteritis in suckling piglets, characterized by vomiting, severe watery diarrhea, and dehydration, and is associated with high mortality.

In recent years, the risk of animal-derived coronaviruses infecting humans has garnered global attention. SARS-CoV, MERS-CoV, COVID-19 (also known as SARS-CoV-2), and seasonal HCoVs are all considered to have originated in animals and have infected humans via bats or intermediate hosts [12]. These cases demonstrate that cross-species spillover from animals to humans is a realistic public health threat. Alphacoronavirus suis viruses such as FCoV, CCoV, and TGEV infect animals that frequently come into contact with humans, posing a potential public health risk. Viruses similar to CCoV and FCoV have recently been detected in humans [13,14,15], suggesting cross-species transmission within this virus group. Known human-infecting Alphacoronaviruses, such as HCoV-229E and HCoV-NL63, belong to different subgenera within the same genus and are phylogenetically distinct. Therefore, if a virus belonging to *Alphacoronavirus suis* were to infect humans, it would represent cross-species transmission from a novel subgenus, potentially posing a new public health risk.

The development and identification of effective antiviral drugs are crucial strategies for controlling viral infections. Molnupiravir is a prodrug of the synthetic nucleoside N4-hydroxycytidine (NHC) and exerts antiviral effects by targeting RNA-dependent RNA polymerase (RdRp) [16,17]. Its active metabolite, NHC triphosphate (NHC-TP), acts as a pyrimidine analog and induces error catastrophe by randomly incorporating adenine or guanine at purine positions in viral genome RNA, thereby irreversibly inhibiting viral replication.

Previous studies reported that molnupiravir exerted antiviral effects against FCoV both in vitro and in vivo [18,19,20,21,22]. Therefore, it has potential as an antiviral candidate against viruses derived from *Alphacoronavirus suis*. Although molnupiravir has demonstrated antiviral activity against other alphacoronaviruses, including HCoV-229E and HCoV-NL63 [23], its efficacy against viruses classified under *Alphacoronavirus suis*, including CCoV and TGEV, has not yet been investigated in detail. Therefore, the present study examined the in vitro antiviral effects of molnupiravir against FCoV, CCoV, and TGEV to assess its efficacy against viruses belonging to *Alphacoronavirus suis*.

To ensure consistency in evaluating the antiviral effects of molnupiravir, we utilized the fcwf-4 cell line for all Alphacoronavirus suis strains tested. Although fcwf-4 cell line is derived from feline tissue and not species-specific for canine or porcine viruses, previous studies have demonstrated its permissiveness to FCoV-2, CCoV-2, and TGEV via the feline aminopeptidase N (fAPN) receptor [24], and to FCoV-1 via an unknown receptor [25,26,27]. Additionally, the intracellular metabolism of molnupiravir and its active metabolite NHC can vary depending on the expression levels of enzymes such as uridine–cytidine kinases (UCK), whose levels differ among cell lines [28]. By using a single cell line, we aimed to minimize variability in drug metabolism and ensure uniform assessment of antiviral activity across all tested viruses.

## 2. Materials and Methods

### 2.1. Cell Cultures

fcwf-4 cells were kindly provided by Dr. M. C. Horzinek of Utrecht University, the Netherlands. Cells were cultured in Eagle’s minimum essential medium supplemented with 50% L-15 medium, 100 U/mL of penicillin, and 100 U/mL of streptomycin. Fetal bovine serum was added at a concentration of 5% for growth medium and 2% for maintenance medium (MM).

### 2.2. Viruses

Two FCoV strains, one CCoV strain, and one TGEV strain, all classified under *Alphacoronavirus suis*, were used in the present study. The FCoV-1 strain KU-2 was previously isolated in our laboratory. The FCoV-2 strain 79-1146 was kindly provided by Dr. M. C. Horzinek of Utrecht University, the Netherlands. The CCoV-2 strain 1-71 was kindly provided by Dr. E Takahashi of the University of Tokyo. The TGEV strain TO-163 was obtained from the National Institute of Animal Health of Japan. All viruses were propagated in fcwf-4 cells at 37 °C. The GenBank accession numbers of the viral strains used in this study are as follows: FCoV-1 strain KU-2 (LC880185.1), FCoV-2 strain 79-1146 (AY994055.1), CCoV-2 strain 1-71 (AY796289.1), and TGEV strain TO-163 (AB115401.1).

### 2.3. Compounds

Molnupiravir was obtained from Cayman Chemical Company (Ann Arbor, MI, USA) and dissolved in phosphate-buffered saline [PBS(−)] to a concentration of 10 mM. In all experiments, except cytotoxicity assays, PBS(−) at the same final concentration as the highest dose of molnupiravir was used as the vehicle control.

### 2.4. Cytotoxic Effects of Compounds

fcwf-4 cells were seeded at a density of 4 × 10^5^ cells in 100 μL of MM on a 96-well plate and were incubated at 37 °C for 24 h. After the incubation, the supernatants were removed, and molnupiravir was added to the remaining cells. Cells were then incubated for an additional 24 h and subsequently washed. Water-soluble tetrazolium salt-8 (WST-8, Kishida Chemical Co., Ltd., Osaka, Japan), diluted 10-fold with MM, was added at a volume of 100 µL per well. Following an incubation at 37 °C for 1 h, absorbance was measured at 450 and 650 nm using a microplate reader. Percent cytotoxicity was calculated using the following formula: Cytotoxicity (%) = [(OD of untreated cells − OD of treated cells)/(OD of untreated cells)] × 100. This cytotoxicity assay was conducted using the same methodology as in previous studies [29,30,31]. Based on the ability to quantify concentration-dependent cytotoxic effects of solvents and compounds, a positive control was not included in each assay.

### 2.5. Plaque Reduction Assay

fcwf-4 cells were cultured as a monolayer in 24-well plates and inoculated with 100 µL/well of a virus suspension containing 7 × 10^2^ PFU/mL. After 1 h of adsorption at 37 °C, the virus suspension was removed, and cells were washed with FBS-free E-MEM. An overlay medium consisting of 1.5% carboxymethyl cellulose in MM, containing serial two-fold dilutions of molnupiravir, was added at 1 mL/well. FCoV-1 was incubated for 72 h, whereas FCoV-2, CCoV-2, and TGEV were incubated at 37 °C for 48 h. After the incubation, cells were fixed and stained with 1% crystal violet in 10% neutral buffered formalin for 30 min. The number of plaques was counted, and the plaque reduction rate was calculated using the following formula:

Plaque Reduction Rate (%) = [(Number of plaques in untreated cells − Number of plaques in treated cells)/Number of plaques in untreated cells] × 100

### 2.6. Molnupiravir Treatment and Sample Collection

fcwf-4 cells were cultured as a monolayer in 24-well plates and inoculated with 100 µL/well of the virus suspension containing 7 × 10^2^ PFU/mL. After 1 h of adsorption at 37 °C, the virus suspension was removed, and cells were washed with FBS-free E-MEM. Serial dilutions of molnupiravir in MM were added at 1 mL/well. FCoV-1 was incubated for 72 h, while FCoV-2, CCoV-2, and TGEV were incubated at 37 °C for 48 h. After the incubation, culture fluids containing cells and supernatants were collected and centrifuged at 3000 rpm at 4 °C for 5 min. The resulting supernatants were collected as culture supernatant samples. Cell pellets were washed three times with PBS(–) and stored at −80 °C. Uncentrifuged culture fluids were also stored at −80 °C as whole-culture samples.

### 2.7. Virus Titration

Cell samples were supplemented with 1 mL of MM to match the volume of the culture supernatant. MM-supplemented cell samples and whole-culture samples, which contained cellular debris, were both centrifuged at 3000 rpm at 4 °C for 5 min. The resulting supernatants of all sample types (culture supernatant, cell, and whole-culture samples) were then serially diluted ten-fold in MM and inoculated into fcwf-4 cells cultured as a monolayer in 96-well plates. After an incubation for 48 or 72 h, cytopathic effects were assessed. The 50% tissue culture infectious dose (TCID_50_) was calculated using the Reed–Muench method.

### 2.8. Quantification of Intracellular FCoV 3′-UTR Expression

Total cellular RNA was extracted from fcwf-4 cell samples using the High Pure RNA Isolation Kit (Roche Diagnostics GmbH, Mannheim, Germany) according to the manufacturer’s instructions. RNA was eluted in 50 μL of elution buffer. The quantification of FCoV 3′-UTR was performed with RT-qPCR using a primer set and probe as previously described by Gut et al. [32]. Reverse transcription and amplification were conducted using the RNA-direct Realtime PCR Master Mix (TOYOBO, Osaka, Japan) with the following oligonucleotides: forward primer (5′-GATTTGATTTGGCAATGCTAGATTT-3′), reverse primer (5′-AACAATCACTAGATCCAGACGTTAGCT-3′), and probe (FAM-5′-TCCATTGTTGGCTCGTCATAGCGGA-3′BHQ1). Reactions were performed in a total volume of 20 μL per well in 48-well PCR plates using the StepOne Real-Time PCR System (Thermo Fisher Scientific, MA, USA). Thermal cycling conditions were as follows: at 90 °C for 30 s, 60 °C for 20 min, and 95 °C for 1 min, followed by 45 cycles at 90 °C for 15 s and 60 °C for 1 min. The absolute quantification of FCoV 3′-UTR copy numbers was performed using an RNA standard curve as previously described by Doki et al. [33]. The reduction in intracellular FCoV 3′-UTR levels was calculated using the following formula: Intracellular FCoV 3′-UTR Reduction (%) = [(copy number in untreated cells − copy number in treated cells)/(copy number in untreated cells)] × 100.

### 2.9. Indirect Immunofluorescence Assay (IFA) for the Detection of Viral Antigens

IFA was performed using the anti-FCoV N protein monoclonal antibody (mAb) Y-N-2, previously described by Takano and Hohdatsu [34], as the primary antibody. Since mAb Y-N-2 does not react with the N protein of CCoV-2, mAb Y-N-1, developed in the same study, was used for CCoV-2 detection. The specificity of anti-FCoV N protein mAbs was confirmed through Western blotting (Figure S1).

fcwf-4 cells were cultured as a monolayer in 96-well plates and inoculated with 100 µL/well of the virus suspension containing 7 × 10^2^ PFU/mL. After 30 min of adsorption at 37 °C, the cells were washed and treated with 100 µL/well of serially diluted molnupiravir in 2% E-MEM. FCoV-2 was incubated for 19 h, while FCoV-1, CCoV-2, and TGEV were incubated at 37 °C for 48 h. After the incubation, the supernatants were removed and cells were washed three times with PBS(–). Cells were then fixed with 100 µL/well of 2% formaldehyde (Nacalai Tesque, Japan) in PBS(–) for 20 min at room temperature. After washing, 100 µL/well of methanol was added, and the plates were incubated at −20 °C for 20 min. Following methanol removal and air drying, cells were incubated with the respective primary mAbs at 37 °C for 60 min: mAb Y-N-2 was used for FCoV-1, FCoV-2, and TGEV, while mAb Y-N-1 was used for CCoV-2. After washing with PBS(–), cells were incubated with the FITC-conjugated anti-mouse IgG (H+L) F(ab’)_2_ fragment as the secondary antibody at 37 °C for 60 min. After a final wash with PBS(–), 100 µL/well of DAPI staining solution was added and incubated for 20 min. Cells were then washed and 100 µL/well of PBS(–) was added prior to observations under an inverted fluorescence microscope (ECLIPSE Ts2, Nikon Corporation, Tokyo, Japan).

### 2.10. Statistical Analysis

Multiple comparisons between treatment groups and the control group were performed using Dunnett’s test. Multiple comparisons among all groups were performed using Tukey’s HSD test. All statistical analyses were conducted using JMP^®^ Pro version 18.0.2 (SAS Institute Inc., Cary, NC, USA). In this study, a *p*-value of less than 0.05 was considered statistically significant.

## 3. Results

### 3.1. Cytotoxicity of Molnupiravir in fcwf-4 Cells

**Figure 1:**
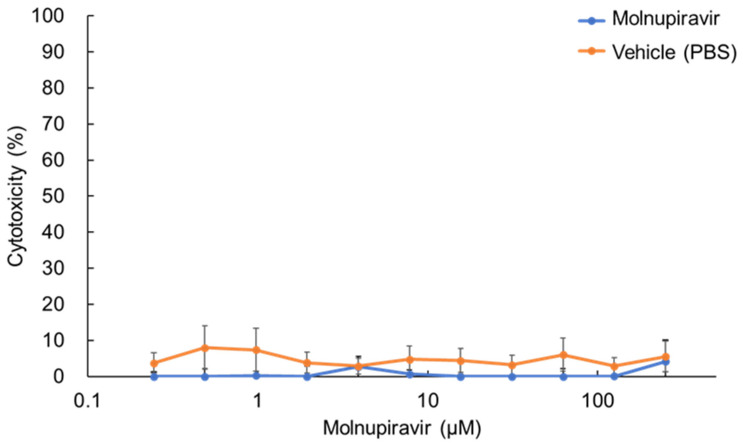
Cytotoxic effects of molnupiravir on fcwf-4 cells. Cells were treated with various concentrations of molnupiravir for 24 h. Cytotoxicity was assessed using the WST-8 assay. Percent cytotoxicity was calculated using the following formula: Cytotoxicity (%) = [(OD of untreated cells − OD of treated cells)/OD of untreated cells] × 100. Results are presented as means ± SE. Data represent three independent experiments.

The cytotoxic effects of molnupiravir on fcwf-4 cells were evaluated after 24 h of treatment across a concentration range from 0.245 to 250 µM. No concentration-dependent increase in cytotoxicity was observed (Figure 1). Even at the highest concentration tested (250 µM), molnupiravir exhibited minimal cytotoxicity, with a cytotoxicity rate of only 4.1%. Similarly, the vehicle control (PBS) showed no concentration-dependent cytotoxic effects.

### 3.2. Plaque Reduction Activity of Molnupiravir Against Alphacoronavirus suis

**Figure 2:**
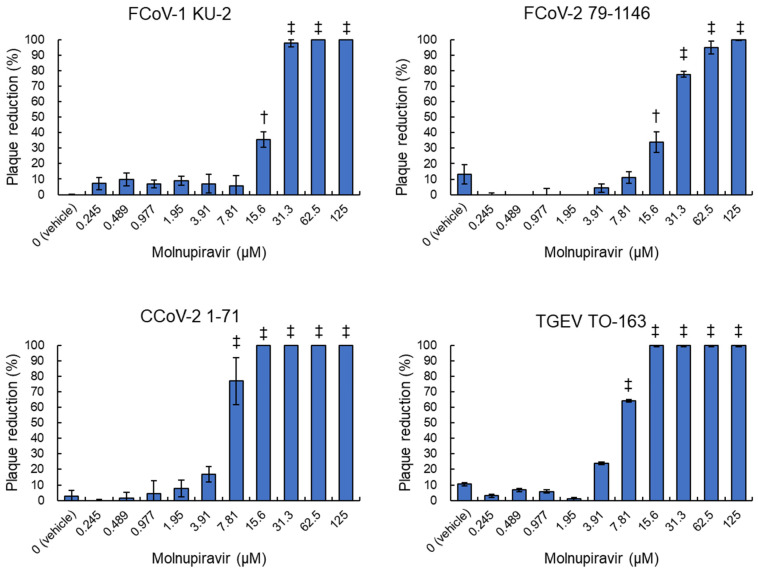
Inhibitory effects of molnupiravir on plaque formation induced by *Alphacoronavirus suis*. fcwf-4 cells were infected with *Alphacoronavirus suis* and cultured in an overlay medium containing serial dilutions of molnupiravir. The plaque reduction rate was calculated using the following formula: Plaque Reduction Rate (%) = [(Number of plaques in untreated cells − Number of plaques in treated cells)/Number of plaques in untreated cells] × 100. †: *p* < 0.05 vs. vehicle. ‡: *p* < 0.01 vs. vehicle. Results are presented as means ± SE. Data represent three independent experiments.

fcwf-4 cells were infected with four alphacoronavirus strains: FCoV-1 KU-2, FCoV-2 79-1146, CCoV-2 1-71, and TGEV TO-163, and then treated with serial dilutions of molnupiravir. The number of plaques that formed was used to calculate the plaque reduction rate. Molnupiravir significantly inhibited plaque formation in a dose-dependent manner across all virus strains (*p* < 0.05 or *p* < 0.01, Figure 2). Complete inhibition (100%) was observed at concentrations ≥62.5 µM for FCoV-1 and ≥15.6 µM for both CCoV-2 and TGEV. Regarding FCoV-2, plaque formation was nearly completely inhibited (99.8%) at the highest tested concentration of 125 µM.

Calculated IC_50_ values were 17.8 µM for FCoV-1, 20.9 µM for FCoV-2, 6.1 µM for CCoV-2, and 6.6 µM for TGEV (Table 1). Notably, statistical analysis confirmed significant differences in IC50 values between FCoV strains and CCoV-2/TGEV. No plaque reduction was observed in vehicle-treated controls.

### 3.3. Inhibitory Effects of Molnupiravir on Viral Replication

**Figure 3:**
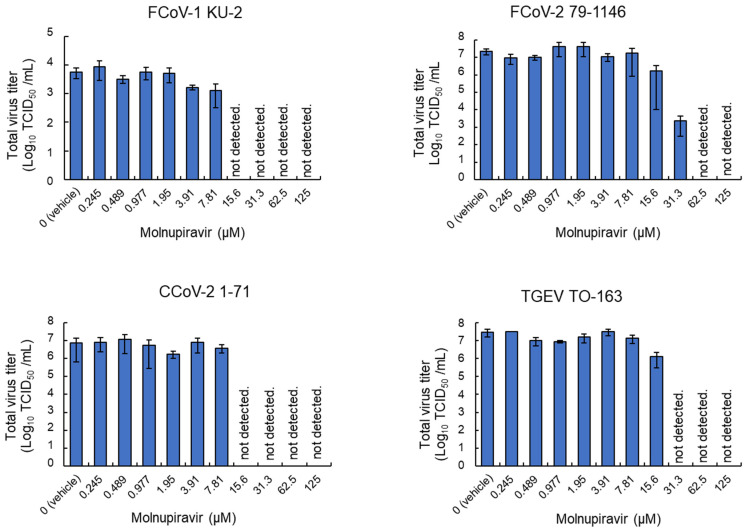
Molnupiravir-mediated reductions in infectious virus titers in whole-culture samples. fcwf-4 cells were infected with *Alphacoronavirus suis* and cultured in MM containing serial dilutions of molnupiravir. Whole-culture samples, including both cells and supernatants, were collected after the incubation. Total virus titers in samples were assessed using the TCID_50_ method. Results are presented as means ± SE. Data represent three independent experiments.

To examine the antiviral effects of molnupiravir on viral replication, whole-culture samples were collected from infected fcwf-4 cells and subjected to virus titration. Regarding FCoV-1 and CCoV-2, infectious virus particles were undetectable at concentrations ≥15.6 µM (Figure 3). Furthermore, no infectious particles were detected for TGEV at ≥31.3 µM. In contrast, FCoV-2 showed a reduction in the viral titer at 31.3 µM and complete inhibition at concentrations ≥62.5 µM. No significant reduction in viral titers was observed at concentrations ≤7.81 µM for any strain.

### 3.4. Suppression of Viral Antigen Expression by Molnupiravir

**Figure 4:**
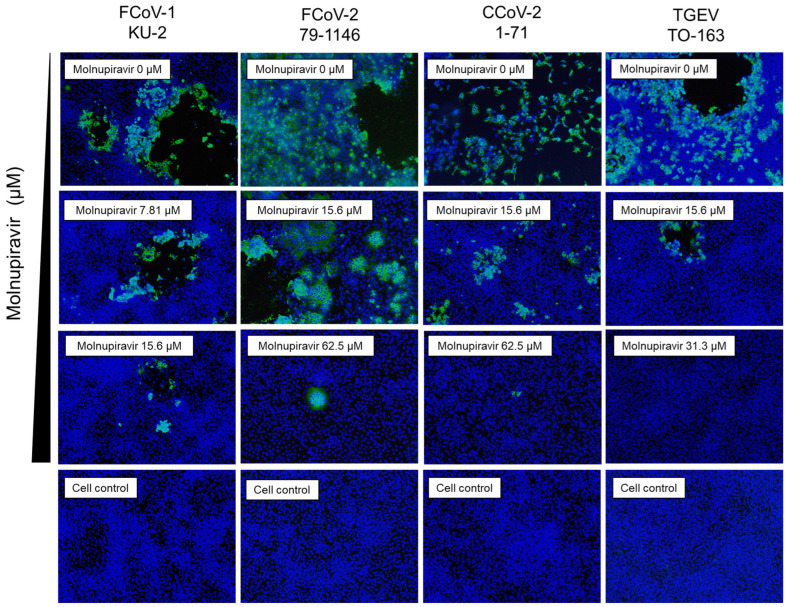
Immunofluorescence analysis of the molnupiravir-mediated suppression of viral antigen expression in fcwf-4 cells infected with *Alphacoronavirus suis*. fcwf-4 cells were infected with *Alphacoronavirus suis* and treated with molnupiravir. After the incubation, cells were fixed and stained with an anti-N protein antibody (green) to detect viral antigen expression and with DAPI (blue) to visualize cell nuclei. Representative immunofluorescence images are shown for each treatment condition.

Immunofluorescence staining was performed to evaluate the expression of the viral N protein in infected fcwf-4 cells. The molnupiravir treatment resulted in a concentration-dependent reduction in N protein-positive cells for all tested viruses (Figure 4). The minimum concentrations at which a clear reduction was observed were 15.6 µM for FCoV-1, 62.5 µM for FCoV-2 and CCoV-2, and 31.3 µM for TGEV. It should be noted that the immunofluorescence assay was performed under high MOI conditions using a 96-well plate format, which differs from the low MOI conditions used in plaque and virus titration assays. This may account for the detection of N protein-positive cells despite the absence of infectious virus particles.

### 3.5. Molnupiravir Inhibits Viral Release and Genome Replication

**Figure 5:**
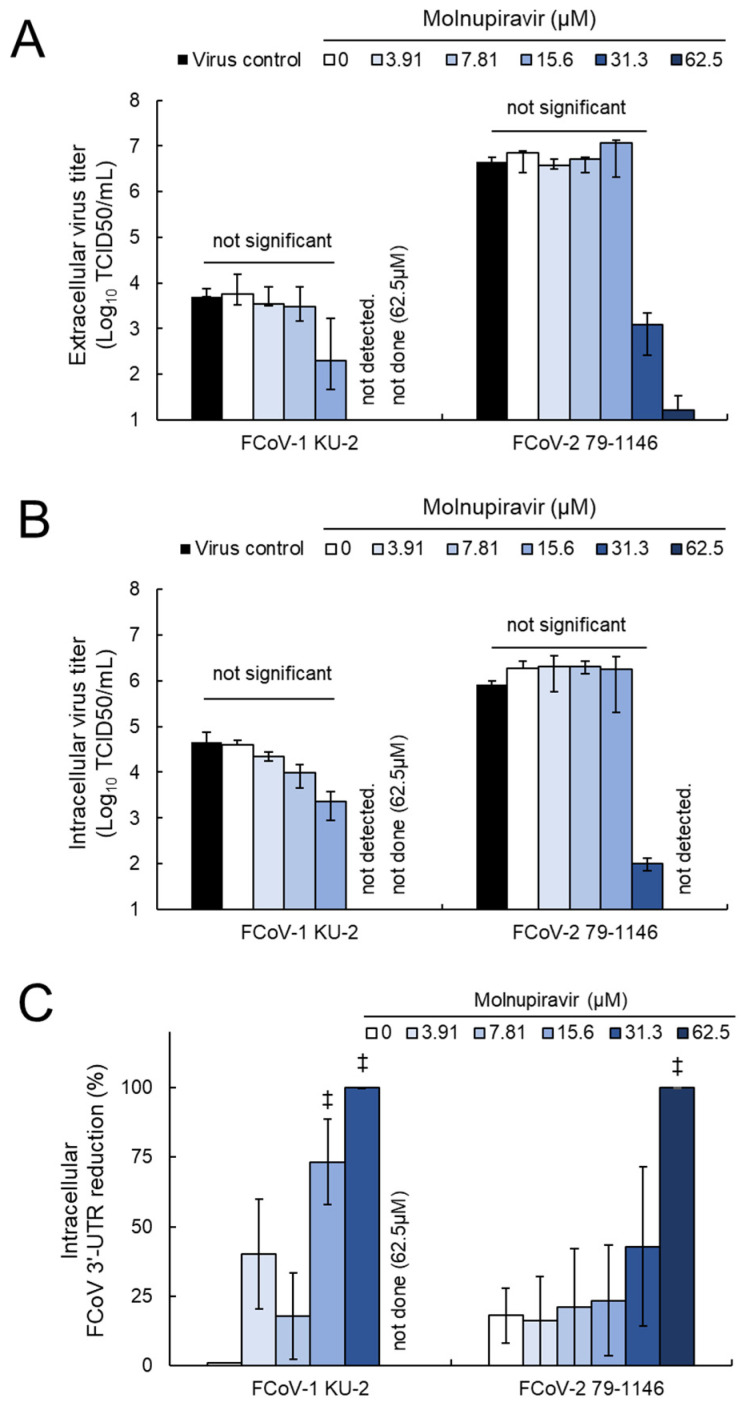
Molnupiravir inhibits viral release and replication in fcwf-4 cells infected with *Alphacoronavirus suis*: (**A**) Extracellular virus titers, (**B**) intracellular virus titers, and (**C**) intracellular FCoV 3′-UTR expression levels were quantified in fcwf-4 cells infected with *Alphacoronavirus suis* and treated with molnupiravir. Virus titers were assessed using the TCID_50_ method, and viral RNA levels were measured using RT-qPCR. ‡: *p* < 0.01 vs. vehicle. Results are presented as means ± SE. Data represent three independent experiments.

To further investigate the underlying mechanism of action, extracellular and intracellular virus titers and intracellular viral RNA levels were quantified in fcwf-4 cells infected with FCoV-1 or -2. The extracellular fraction was separated from the cell pellet via centrifugation after incubation. The cell pellet was washed and subjected to freeze–thaw cycles. The resulting cell lysate was divided into two portions: one for virus titration and the other for RNA extraction. These samples were treated as the intracellular fraction and intracellular FCoV RNA, respectively. Infectious virus particles for FCoV-1 were undetectable in both the extracellular and intracellular fractions at concentrations ≥15.6 µM (Figure 5A,B). The viral titers of FCoV-2 were reduced at 31.3 µM and undetectable at concentrations ≥62.5 µM. RT-qPCR analysis revealed a statistically significant decrease in intracellular FCoV 3′-UTR expression (*p* < 0.01, Figure 5C). A dose-dependent reduction in intracellular FCoV 3′-UTR levels was observed at concentrations ≥7.81 µM for FCoV-1 and ≥15.6 µM for FCoV-2. These results suggest that molnupiravir inhibits both viral genome replication and the release of infectious virus particles.

Notably, the quantification of viral release and genome replication was limited to FCoV-1 and FCoV-2. FCoV-1 is known to differ from FCoV-2, CCoV-2, and TGEV in that it does not utilize the well-characterized aminopeptidase N (APN) receptor for cell entry, instead relying on an unidentified receptor. This distinction suggests that FCoV-1 may exhibit unique interactions with host cells and replication characteristics. Therefore, a comparative analysis with FCoV-2 was considered essential to better understand the antiviral mechanism of molnupiravir. In contrast, for CCoV-2 and TGEV, sufficient antiviral effects were already demonstrated through viral titration and antigen expression data, and the added value of RNA quantification was deemed limited.

## 4. Discussion

Following the global spread of COVID-19, the search for and evaluation of antiviral drug candidates against coronaviruses has accelerated worldwide. However, only a limited number of drugs have been approved for human use and clinically applied to the treatment of COVID-19. Molnupiravir is an orally administered nucleoside analog with potent antiviral activity against SARS-CoV-2 and is currently used as a COVID-19 therapeutic in many countries [17]. The repurposing of clinically approved antiviral agents for other coronaviruses is a rational and practical approach to prepare for emerging zoonotic threats.

Molnupiravir has also been shown to suppress the replication of FCoV and is used as a treatment for feline infectious peritonitis, a fatal FCoV infection [20,21,22]. Given its efficacy against FCoV, molnupiravir may also be effective against CCoV and TGEV, which belong to the same *Alphacoronavirus suis* species. In the present study, we demonstrated that molnupiravir suppressed plaque formation, viral antigen expression, the production of infectious viral particles, and viral RNA levels in these viruses.

Using fcwf-4 cells to propagate these viruses, IC_50_ values for CCoV-2 and TGEV were significantly lower than those for FCoV-1 and -2, suggesting that these viruses were more sensitive to molnupiravir. This difference may be attributed to differences in viral factors, such as the structure or sequence of RNA-dependent RNA polymerase, among the tested virus species. To date, no coronaviruses have been reported to exhibit resistance to molnupiravir, and the mechanisms underlying sensitivity and resistance remain unclear [35]. NHC-TP is incorporated into the RdRp of coronaviruses, where it induces error catastrophe during viral replication [23]. Therefore, differences in the amino acid sequences or structures of RdRp among virus species may affect the incorporation efficiency of NHC-TP and the resulting mutation rates, potentially explaining the observed differences in sensitivity to molnupiravir.

Additionally, feline-derived fcwf-4 cells were used in the present study, differing from the natural hosts of CCoV-2 and TGEV, namely, dogs and pigs, respectively. This mismatch between the cell type and host species may have affected viral replication efficiency and drug sensitivity. Future studies need to include systematic comparisons using cell lines derived from the natural hosts of CCoV-2 and TGEV. Furthermore, comparative analyses of RdRp gene sequences and in vitro enzymatic activity assays are required to elucidate the molecular basis of molnupiravir sensitivity.

Previous studies demonstrated that molnupiravir exhibited antiviral activity against other Alphacoronaviruses, such as HCoV-229E, HCoV-NL63, and porcine epidemic diarrhea virus [23,36]. These findings and the present results indicate that molnupiravir exhibits broad-spectrum antiviral activity against Alphacoronaviruses that infect both humans and animals. Its efficacy against viruses with potential for cross-species transmission is particularly significant from a One Health perspective.

In conclusion, molnupiravir exhibited broad in vitro antiviral activity against multiple viruses classified under *Alphacoronavirus suis*. These results highlight its potential as an antiviral drug candidate in veterinary medicine and suggest its applicability to the prevention and control of future zoonotic infections.

## Figures and Tables

**Table 1:** Molnupiravir concentration required for 50% viral plaque reduction.

Virus Strains	IC_50_ (μM, 95%CI)
FCoV-1 KU-2	17.8 ^a^ (15.9–19.6)
FCoV-2 79-1146	20.9 ^a^ (18.1–23.6)
CCoV-2 1-71	6.1 ^b^ (5.4–6.8)
TGEV TO163	6.6 ^b^ (6.1–7.2)

^a/b^: Different letters indicate statistically significant differences (*p* < 0.01).

## Data Availability

The datasets generated for this study are available upon request to the corresponding author.

## Supplementary Materials

The following supporting information can be downloaded at: https://www.mdpi.com/article/10.3390/pathogens14080787/s1, Figure S1: Specificity of anti-FCoV N protein monoclonal antibodies (mAbs).
