# Supplementary figures and images for: Molnupiravir Inhibits Replication of Multiple Alphacoronavirus suis Strains in Feline Cells
Source: Pathogens. 2025 Aug 7;14(8):787. doi: 10.3390/pathogens14080787 (PMC12389489; doi:10.3390/pathogens14080787)

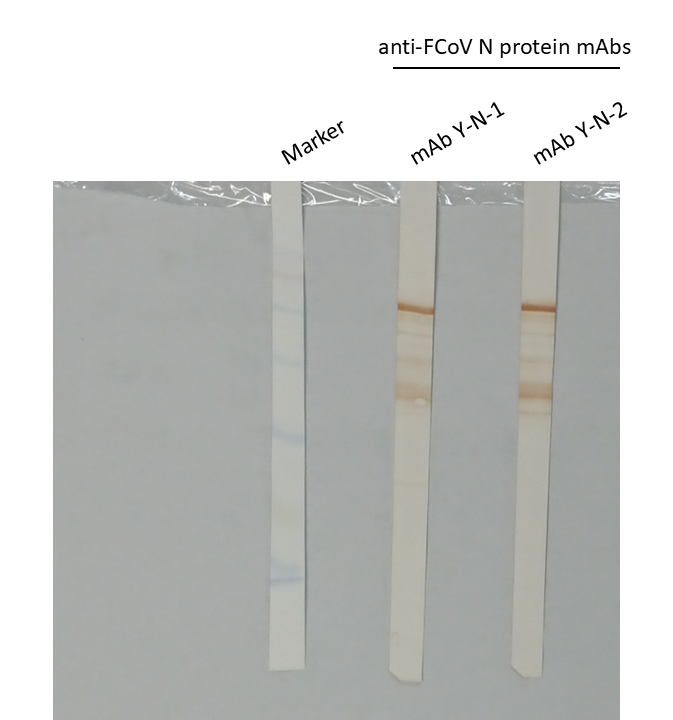

Supplement: Supplementary file 1 [file pathogens-14-00787-s001.zip › pathogens-3723755-supplementary Figure S1.tif]
